# Impact of Health Promotion Interventions on Early Childhood Caries Prevention in Children Aged 2–5 Years Receiving Dental Treatment Under General Anesthesia

**DOI:** 10.3389/fpubh.2020.00006

**Published:** 2020-02-26

**Authors:** Samaneh Razeghi, Pardis Amiri, Simin Z. Mohebbi, Mohammad J. Kharazifard

**Affiliations:** ^1^Research Center for Caries Prevention, Dentistry Research Institute, Tehran University of Medical Sciences, Tehran, Iran; ^2^Department of Community Oral Health, School of Dentistry, Tehran University of Medical Sciences, Tehran, Iran; ^3^Private Practitioner, Mashhad, Iran; ^4^Dental Research Center, Dentistry Research Institute, Tehran University of Medical Sciences, Tehran, Iran

**Keywords:** early childhood caries, general anesthesia, educational intervention, quality of life, oral health promotion

## Abstract

**Aim:** This study was conducted to evaluate the impact of health promotion interventions on early childhood caries prevention in 2–5 year-olds receiving dental treatment under general anesthesia.

**Materials and Methods:** Thirty-seven mother-child couples presenting to the clinic of the Dental School of Tehran University of Medical Sciences for treatment under general anesthesia were randomly divided to two groups: 19 couples in the pamphlet and fluoride varnish four times a year, and 18 couples in the pamphlet plus six phone call reminders and fluoride varnish four times a year. A standard questionnaire on demographics and children oral health-related practice of parents was completed by respondents. On children's oral examination, the Simplified Oral Hygiene Index (OHI-S), dmft, and the presence of new white spot lesions (WS) were recorded in both phases. At the final stage, Early Childhood Oral Health Impact Scale (ECOHIS) was completed by parents. The length of follow-up was 24 months.

**Results:** In both groups, there was an increase in the number of mothers who knew how to brush their children's teeth as well as the number of mothers who brushed their children's teeth (*P* < 0.05). In the reminder group, an improvement occurred in the mothers' perception of their perceived ability to make their children brush their teeth twice a day (*P* = 0.03). Clinical examination revealed a significant decrease in the OHI-S (from 1.9 ± 0.8 to 1.15 ± 0.5) and the number of WS (from 8.5 ± 5.5 to 0.08 ± 0.5) in both groups on the follow-up visit. The mean dmft was 11.0 ± 4.0 with a mean d component of 10.56 ± 4 at the baseline, which decreased significantly to 1.44 ± 1.96 after dental treatment. No significant increase was seen in new caries in the intervention groups. There was no significant difference in the ECOHIS score between the two groups.

**Conclusion:** The similar impact of both interventions suggests the possibility of applying the simpler one, i.e., the educational pamphlet, fluoride varnish and frequent follow-ups. However, in the reminder group, the mothers' perception of their perceived ability to make children brush their teeth twice a day was improved.

## Introduction

The overall prevalence of caries has been decreasing in recent decades although it remains the most common chronic disease in childhood (1, 2). The most recent report on the Iranian oral health showed a high prevalence of dental caries in preschool children (more than 87% of 5–6 year-olds) with a mean dmft of 5.16 and the d component comprising more than 70% of the total mean (3).

Dental caries in preschoolers poses a clear risk to the general health as a common disease in childhood. Additionally, this disease is associated with a marked functional, aesthetic, and psychological impact on the quality of life in children and their families, and imposes high costs on them (2, 4). Moreover, primary teeth caries is the strongest predictor of caries in the permanent dentition (1, 5). Despite development of alternative treatment modalities, many children still require dental treatment under general anesthesia (GA), which is costly and risky (6, 7). Unfortunately, a marked proportion of these children may need further dental treatment under GA due to the disease or problems not present at the time of the first GA (1, 6, 8). Therefore, despite the extreme stress of dental treatment under GA for parents, it does not seem to raise any alarms regarding modification of their children's oral health-related behaviors in the long term (9).

Dental caries is mostly preventable through controlling several factors affecting its onset and progression, such as dietary practices, preventive care, certain drugs (7, 10), and improved oral health literacy (11). Oral health preventive strategies for the whole family, such as educational intervention beside child-focused preventive strategies, have shown to be important for pediatric oral health preservation (4, 12, 13). Oral health education in various aspects such as tooth brushing, dietary and feeding habits, and regular dental visits, together with using fluoride supplementation have been well-documented as useful methods for ECC prevention (12–14).

To our knowledge, however, the effectiveness of various forms of health promotion interventions on ECC prevention, especially for children receiving dental treatment under general anesthesia, has not been investigated. The objective of the present study was to evaluate the long-term effectiveness of two health promotion interventions on early childhood caries prevention in children aged 2–5 years receiving dental treatment under GA and to investigate the oral health-related quality of life (OHRQoL) in parents.

## Materials and Methods

### Sampling

The Minitab software (Minitab Inc., Pennsylvania, USA) was used with a minimum significant difference = 15%, mean standard deviation = 19% for OHI-S, alpha = 0.05, and beta = 0.2 for sample size determination (15). The sample size calculation showed that 18 mother-child pairs were required for the baseline study.

Thirty-seven pairs of mothers and their 2–5 year-old children who needed dental treatment under general anesthesia were selected and randomly divided to two intervention groups: 19 couples in the pamphlet and fluoride varnish four times a year (PV) and 18 couples in the pamphlet plus six phone call reminders and fluoride varnish four times a year (PVR).

### Study Methodology and Interventions

Before dental treatment under GA, the mothers completed an anonymous self-administrated valid and reliable questionnaire (16). Moreover, all children underwent a clinical dental examination. After dental treatment under GA, all mothers received a pamphlet containing recommendations (14) on proper tooth brushing, using appropriate amount of fluoridated tooth paste, proper dietary and feeding habits, and the necessity for regular dental visits and using fluoride varnish. This material was written in Farsi and had colorful pictures and a proper instructional design.

Two years after the interventions, all couples were recalled and the same questionnaire was completed by mothers. Another clinical oral examination was done for all children. During this period, all children had regular dental check-ups with fluoride varnish application every 3 months; moreover, the reminder group received six reminder phone calls once a month during the first 6 months after dental treatment under GA. In these 20 min phone calls, one of the researchers provided oral health recommendations for the child, which matched the educational pamphlet content completely.

### Questionnaire

The mothers were requested to complete a questionnaire before and 2 years after the interventions. They were asked to write a unique code at the top of the first and second questionnaires. This code was used to assess individual changes throughout the study. In addition to demographic characteristics (child's age and gender, birth order, primary caregiver, family income, and parents' level of education), the questionnaire included the following items:

Feeding Habits (Multiple-Choice Questions).

Total duration of prior breastfeeding, total duration of prior bottle-feeding, nighttime feeding practices, most common contents of daytime bottle, frequency of giving sugary snacks.

Mother's and Child's Oral Cleaning Habits (Multiple-Choice Questions).

Cleaning frequency (for both mother and child), cleaning device (for child), and adult's role in oral cleaning for the child.

Mother's Perceptions of Her Ability to Maintain the Child's Oral Hygiene (a 5-Point Likert Scale Ranging From Strongly Agree to Strongly Disagree).

Three statements regarding knowing how to brush the child's teeth properly, devoting sufficient time to brushing child's teeth, and having the ability to make the child brush his/her teeth twice a day.

Besides, in the 2 year recall, all mothers completed the Persian version of the Early Childhood Oral Health Impact Scale (ECOHIS) containing 13 questions on OHRQoL based on the perceptions of parents and their understanding of health and illness of their children (17, 18). This questionnaire has two components: nine questions about the impacts of oral health on the child's daily activities and 4 questions about these impacts on the family. Each question assesses the frequency of an oral health-related problem and is scored from 1 (never) to 5 (very often) with a choice of “I don't know.” The possible range of ECOHIS score is 13–65 with higher scores indicating greater impacts and/or more problems.

### Oral Examination

An experienced pediatric dentist conducted the oral examination of children in both stages. Using a dental mirror and explorer, the children's oral and dental condition was recorded before the beginning of the dental treatment. The Simplified Oral Hygiene Index (OHI-S), the dmft index (the number of decayed, missing, and filled primary teeth), and tooth surfaces with white spot lesions (WS) were recorded. Two years after treatment under GA, all children were re-examined using the above indices. All parents received verbal oral health recommendations in the same sessions.

### Statistical Analysis

Descriptive statistics and the SPSS package were used. Wilcoxon Signed Ranks and Mann–Whitney test were applied to compare the outcome variables between the two groups before and after the interventions. The level of significance was set at 0.05.

## Results

Thirty-seven (19 pairs in PV and 18 pairs in PVR group) and 34 mother-child pairs (18 pairs in PV and 16 pairs in PVR group) attended the baseline and 2 year follow-up data collection for questionnaire completion and oral examination, respectively. The total response rate was 91.9%.

At baseline data collection, the mean age of the children was 46.5 ± 10.7 months (44.6 ± 10.3 months in PV and 48.5 ± 11.1 months in PVR) of whom 24 (64.9%) were boys and 18 (48.6%) were first children regarding the birth order. In most of the children (94.6%), the mothers were the main caregivers and the others went to kindergarten. Table 1 shows the demographic characteristics of the participants in both groups.

**Table 1:** Demographics of study participants (*n* = 37).

**Demographic variables**	**Groups**	
	**PV**	**PVR**
Child gender		
Boy	13 (68.4%)	11 (61.1%)
Girl	6 (31.6%)	7 (38.9%)
Birth order		
First	11 (57.9%)	7 (38.9%)
Second/third	8 (42.1%)	8 (44.4%)
Other	0 (0.0%)	3 (16.7%)
Primary caregiver		
Mother	17 (89.4%)	18 (100.0%)
Kindergarten	2 (10.6%)	0 (0.0%)
Family income^*^		
High	4 (25.0%)	3 (23.1%)
Moderate	10 (62.5%)	2 (15.4%)
Low	2 (12.5%)	8 (61.5%)
Mother's level of education		
High	9 (47.4%)	3 (16.7%)
Moderate	9 (47.4%)	12 (66.7%)
Low	1 (5.3%)	3 (16.7%)
Father's level of education		
High	10 (52.6%)	8 (44.4%)
Moderate	5 (26.3%)	5 (27.8%)
Low	4 (21.1%)	5 (27.8%)

* *There were few participants who did not respond to this question*.

### Feeding Habits

The mean total duration of prior breastfeeding was 20.0 ± 9.8 months. Moreover, the mean of total duration of prior bottle-feeding was 15.5 ± 15.3 months. Nighttime breastfeeding in the past was reported by 41.2% of the mothers, and 32.4% of mothers stated that they used to give a bottle containing milk or sweet liquids to their children during the night. Only eight mothers reported that they did not practice night nursing. The most common content of the daytime bottle was cow's milk (23.5%) and daytime sugary snacking was more than twice daily in 64.7% of the children.

On the 2 year follow-up, no significant difference was seen in feeding habits between the two groups (*P* > 0.05). At the same time, in both groups, the total duration of bottle-feeding decreased significantly compared to the baseline (*P* = 0.04). There was no significant change in other feeding habits in each group.

### Mother's and Child's Oral Cleaning Habits

On the 2 year follow-up, 58.8% of the mothers reported brushing their own teeth at least once a day, and 32.4% reported brushing more than once a day. Oral cleaning once a day was reported in 67.4% of all children. The mothers reported oral cleaning twice daily in 26.5% of the children, and only two mothers reported a lower frequency of tooth brushing for their children. In most of the children (91.2%) the most common cleaning device was a toothbrush. In 50% of the children, tooth brushing was done by or with the help of one of the parents.

On the 2 year follow-up, an increase was observed in the number of mothers who brushed their children's teeth in both groups (*P* < 0.05).

### Mother's Perceptions of Her Ability to Maintain the Child's Oral Hygiene

On the 2 year follow-up, 70.6% of the mothers stated that they knew how to brush their children's teeth, and 64.7% stated that they devoted sufficient time to brushing their children's teeth. Moreover, 64.7% of the mothers believed that they could make their children brush their teeth twice a day (Table 2).

**Table 2:** Results of Wilcoxon signed rank test of changes in responses of mothers (*n* = 34) regarding perceptions of their ability to maintain the child's oral hygiene during 2-year follow-up.

		**PV** **N (%)**			**PVR** **N (%)**		
**Mother's perceptions of her ability to maintain the child's oral hygiene**	**Choices**	**Baseline 19 (100.0%)**	**2-year follow-up 18 (100.0%)**	***P*-value**	**Baseline 18 (100.0%)**	**Baseline 19 (100.0%)**	**2-year follow-up 18 (100.0%)**
I don't know how to brush or clean my child's teeth properly	Completely agree	1 (5.3%)	3 (16.7%)	0.03^*^	1 (5.6%)	0 (0.0%)	0.007^*^
	Agree	6 (31.3%)	2 (11.1%)		6 (33.3%)	3 (20.0%)	
	Disagree	5 (26.3%)	10 (55.6%)		7 (38.9%)	8 (53.3%)	
	Completely disagree	4 (21.1%)	3 (16.7%)		1 (5.6%)	3 (20.0%)	
	Do not know	3 (15.8%)	0 (0.0%)		3 (16.7%)	1 (6.7%)	
We don't have time to brush or clean our child's teeth twice daily	Completely agree	1 (5.3%)	2 (11.1%)	0.49	1 (5.6%)	1 (6.3%)	0.42
	Agree	2 (10.5%)	5 (27.8%)		5 (27.8%)	4 (25.0%)	
	Disagree	12 (66.7%)	8 (44.4%)		9 (50.0%)	8 (50.0%)	
	Completely disagree	3 (15.8%)	3 (16.7%)		1 (5.6%)	3 (18.8%)	
	Do not know	1 (5.3%)	0 (0.0%)		2 (11.1%)	0 (0.0%)	
We cannot make our child brush or clean his/her teeth twice daily	Completely agree	3 (15.8%)	5 (29.4%)	0.59	2 (11.1%)	1 (6.3%)	0.03^*^
	Agree	11 (57.9%)	4 (23.5%)		8 (44.4%)	1 (6.3%)	
	Disagree	5 (26.3%)	8 (47.1%)		5 (27.8%)	11 (68.8%)	
	Completely disagree	0 (0.0%)	0 (0.0%)		1 (5.6%)	3 (18.8%)	
	Do not know	0 (0.0%)	0 (0.0%)		2 (11.1%)	0 (0.0%)	

* *P < 0.05*.

Compared to the baseline, no significant difference was seen in the mothers' perceptions of their ability to maintain the child's oral hygiene between two groups (*P* > 0.05). Moreover, in both groups, an improvement occurred in the number of mothers who knew how to brush their children's teeth. In the reminder group, the mothers' perception of their perceived ability to make the children brush their teeth twice a day improved significantly (*P* = 0.03).

### OHRQoL

The mean ECOHIS score was 29.2 in PV, and 28.5 in PVR (from up to 60). The mean ECOHIS score was 18.5 and 16.8 (from up to 45) in the child impact section in PV and PVR, respectively. Moreover, the mean ECOHIS score was 10.7 and 11.7 (from up to 20) in PV and PVR in family impact section, respectively. No significant difference was seen in the mean scores of ECOHIS, child impact section, and family impact section between the two groups.

### Oral Health Status

#### dmft

At baseline, the mean dmft was 11.0 ± 4.0 in all subjects, 11.3 ± 3.6 in PV, and 10.7 ± 4.5 in PVR. In the 2 year follow-up, the mean dmft was 10.1 ± 3.4 in all subjects,10.3 ± 3.7 in PV, and 9.8 ± 3.1 in PVR, indicating no significant difference compared to baseline (*P* = 1.8). Totally, the d component decreased significantly from 10.6 ± 4.3 at baseline to 1.4 ± 1.96 in the follow-up visit after dental treatments.

#### OHI-S

At baseline, the mean OHI-S was 1.9 ± 0.8 in all subjects, 2.0 ± 0.6 in PV, and 1.8 ± 0.9 in PVR. In the 2 year follow-up, the mean OHI-S was 1.2 ± 0.5 in all subjects, 1.1 ± 0.5 in PV, and 1.2 ± 0.4 in PVR. A significant decrease was observed in OHI-S in the 2 year follow-up in both groups.

#### WS

At baseline, the mean WS was 8.5 ± 5.5 in all subjects, 3.8 ± 2.6 in PV, and 4.7 ± 2.9 in PVR. In the 2 year follow-up, the mean WS significantly decreased to 0.1 ± 0.5 in all subjects, 0.2 ± 0.5 in PV, and 0.0 in PVR (*P* < 0.001). No significant difference was observed in the number of WS between the two groups (*P* = 0.17).

## Discussion

The aim of this study was to investigate the long-term impacts of two health promotion interventions on ECC prevention in children aged 2–5 years who underwent dental treatment under general anesthesia. Furthermore, the oral health quality of life was evaluated in the sample population. The results of the study showed an increase in the number of mothers who knew how to brush their children's teeth and the number of mothers who brushed the children's teeth in both interventional groups. The reminder group experienced an improvement in the mothers' perception of their perceived ability to make the children brush their teeth twice a day. Moreover, clinical examination showed a significant decrease in OHI-S and the number of WS in both groups in the follow-up visit. Furthermore, no significant increase was observed in new caries in both groups. The mean ECOHIS score was almost good in both groups. There was no significant difference in the ECOHIS score and its components between the two groups.

General anesthesia is an essential and reliable approach for safe and successful dental treatment in some challenging children in whom behavior guidance techniques are inadequate for their management, e.g., very young children and those who are not cooperative (19). In spite of comprehensive dental treatment under GA, the arte of caries experience is reported to be high in children leading to a second round of treatment under GA in many cases. (9, 19–23). To avoid the development of new caries and repetition of GA, the present study focused on pairs of mothers and their children who received comprehensive dental treatment under GA. On the other hand, parental beliefs and behaviors affect childhood oral health. Many studies investigating health promotion interventions in preschool children have engaged parents because of their critical role and influence in development of proper oral health related behaviors (4, 7, 9, 16, 24–26).

In the final stage of the present study, the mean value of the d component of dmft was 1.4, which was a promising outcome. Furthermore, the mean WS was about 0.1, which confirmed the positive effects of interventions on the prevention of dental caries recurrence. It should be mentioned that nearly all participants were recalled every 3 months for oral examination, fluoride varnish therapy, and oral health consultation. All mothers received verbal oral health recommendations on tooth brushing, daily sugar intake, and feeding habits. These findings are similar to other studies that showed positive impacts of health promotion interventions on ECC prevention (4, 26, 27). This is an encouraging outcome when we notice that many previous studies reported high relapse rates of dental caries after dental treatment under GA in children (9, 19–23). The dmft index decreased in the 2 year follow-up, which was because the age of the children matched early mixed dentition. This might be because of natural exfoliation of some primary teeth.

The OHI-S index decreased significantly in both groups in the 2 year follow-up. Thus, the interventions seem to be successful in promoting oral hygiene status in children. Considering the increased number of mothers who brushed their children's teeth themselves and the number of mothers who knew how to brush their children's teeth, the OHI-S drop was expectable. These findings are consistent with the oral health education goals defined as acquiring knowledge and positive attitude toward oral health, and ultimately adoption of healthy behaviors leading to improved oral health-related indicators (27).

The results of our study showed no significant differences in the study outcomes except for mothers' perception of their ability to make the children brush their teeth twice a day between the two interventional groups, which was better in reminder group. However, in both groups, interventions led to a significant improvement in the number of mothers who brushed their children's teeth and the number of mothers who knew how to brush their children's teeth. The reason for these findings can be the point that the educational content designed for the telephone reminder completely matched the educational content of the pamphlet. Moreover, in regular follow-ups, oral health recommendations were verbally delivered to all mothers in addition to fluoride varnish therapy for all children. It seems that communication with mothers at regular follow-ups and through phone calls promoted oral health behaviors at least in the children's oral cleaning habits as well as the mothers' perception of their ability to maintain the children's oral hygiene. Regular contact with mothers of preschool children for dental health education was supported in a previous study as it showed more preventive effects on dental caries in children when compared to less frequent contacts (28). Besides, the intense emotional effect of GA on parents should be considered as it could motivate them to take immediate action and implement changes in oral health behaviors. However, Amin et al. stated that such an effect would fade over time, leading to poor oral health behaviors in long term (9).

Feeding practices have been target points for interventions for ECC prevention, especially in early stages of life. Early-life feeding habits such as food-intake frequency, breastfeeding, bottle-feeding, and the introduction of complementary foods would affect health over the course of the life (28). Frequent intake of sugars, repeated bottle-feeding at night, *ad libitum* breast-feeding, and breastfeeding more than 12 months are particular risk factors for caries development in children (14, 28, 29). In our study, the mean total duration of prior breastfeeding was about 20 months. This finding was in a line with the results of previous studies in Iran (25) and Canada (9). However, Lee et al. reported that three quarters of children received breastfeeding up to 11 months of age in the US (29). Cultural characteristics and religious beliefs, which recommend breastfeeding up to 24 months of age, may indicate the differences between studies. In the present study, other risky behaviors were nighttime breastfeeding and bottle feeding containing milk or sweet liquids reported by about 41 and 32% of mothers, respectively. Furthermore, about 60% of mothers reported their children consumed sugary snack more than twice a day. These are in contrast to the American Academy of Pediatric Dentistry recommendations for preventing ECC, which advises against frequent consumption of sugary liquids and/or solid foods in a baby bottle, *ad libitum* breast-feeding after the first primary tooth begins to erupt, and baby bottle use after 12–18 months (14).

Moreover, the present study found no significant change in dietary habits in both groups 2 years after GA and interventions. This finding is similar to the results of a study by Lee et al. (29) in the US in which less than half of parents stated they made improvements in their children's diet after GA. The reason may be the child growing up and her/his easier access to more cariogenic foods. It should also be considered that the child's diet is affected by the family's dietary habits, which is very difficult to change. Moreover, it seems that the dietary practices are established and maintained in the first 12 months of life (14).

In our study, in the 2 year follow-up, the mean ECOHIS score was relatively favorable in both groups. Regarding OHRQoL in children after dental treatment under GA, a systematic review by Jankauskiene and Narbutaite revealed that oral rehabilitation using GA could lead to immediate improvement in the quality of life in children and their families (30). Furthermore, another study by Gaynor and Thomson suggested that dental treatment of small children using GA was associated with a marked improvement in the OHRQoL (31). Thus, the results of the present study seem to be consistent with the findings of other studies.

The data collection tool was a questionnaire used in a previous study in Iran (16). In the 2 year follow up, a 13-item questionnaire, the ECOHIS, was added to evaluate the OHRQoL in children. The validity and reliability of the Persian version of the ECOHIS was tested by Jabarifar et al. (17). The self-administered nature of the questionnaires encourages the participants to answer more in line with social norms compared to the actual situation, which is referred to as “social desirability” (32). For instance, a study found evidence of social desirability bias in caregiver-reported oral health behaviors (33). Moreover, recall bias should also be considered since more important events to respondents are more likely to be recalled accurately (15). Another limitation of present study was encouraging participants to attend the final follow-up session. Some parents did not attend follow-ups and had irregular presence for recalls. We tried to overcome this problem by providing adequate explanation for parents as well as free oral examination and fluoride varnish therapy. Furthermore, if a child required new, a treatment session was scheduled. However, three pairs of participants dropped out of the study due to living in far locations and difficult access. Moreover, we could have more significant results if we had a larger sample size; on the other hand, the 2 year follow-up, except for three pairs, was the strength of the study.

## Conclusion

Both interventions improved the self-reported performance of mothers and reduced plaques and white spots. Very few new caries developed in the intervention groups that were not significant. Similar impacts of both interventions suggest the possibility of using the simpler one, i.e., the educational pamphlet, fluoride varnish and frequent follow-ups. However, a marked improvement was seen in the mothers' perception of their perceived ability to make the children brush their teeth twice a day as an attitudinal change in the reminder group.

## Data Availability Statement

The datasets generated for this study are available on request to the corresponding author.

## Ethics Statement

This study was carried out in accordance with the recommendations of The Research Ethics Committee of Tehran University of Medical Sciences (code IR.TUMS.DENTISTRY.REC.1397.097) with written informed consent from all subjects. All subjects gave written informed consent in accordance with the Declaration of Helsinki. The protocol was approved by the The Research Ethics Committee of Tehran University of Medical Sciences.

## Author Contributions

SR and SM led the overall study and contributed to study design. SR and PA contributed to the data collection and wrote the manuscript. MK contributed to the research design and the data analysis and interpretation. All authors read, contributed to, and approved the final manuscript.

### Conflict of Interest

The authors declare that the research was conducted in the absence of any commercial or financial relationships that could be construed as a potential conflict of interest.
